# Letter from the Editor in Chief

**DOI:** 10.19102/icrm.2018.090903

**Published:** 2018-09-15

**Authors:** Moussa Mansour


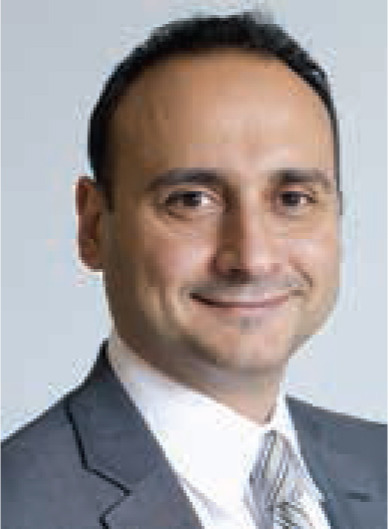


Dear Readers,

Exposure to ionizing radiation has a detrimental effect, yet recent advances in diagnostic and therapeutic tools have led to a sharp increase in the use of radiation to diagnose and treat a variety of conditions. According to the American Society of Radiologic Technologists, between 1980 and 2006, radiation exposure in patients as a result of diagnostic medical imaging increased sixfold.^1^ In one study, patients undergoing myocardial perfusion imaging were believed to have received a cumulative estimated effective dose of more than 100 mSv in just 100 days.^2^ This is a very significant dose knowing that, for patients receiving an effective dose of at least 100 mSv, one in every 100 individuals will develop cancer from a single instance of exposure.^3^ Furthermore, some studies have suggested that there is a correlated increase in the incidence of left-sided brain cancer in physicians who perform invasive catheter-based^4^ or other cardiac procedures.^5^

In this issue of *The Journal of Innovations in Cardiac Rhythm Management*, Kipp et al.^6^ describe a series of 138 electrophysiology study and ablation procedures performed with the intention of not using fluoroscopy. Of the 138 procedures, 105 were successfully performed without fluoroscopy. In the remaining 33 cases (24%), fluoroscopy had to be used for an average of 1.21 minutes ± 1.18 minutes. The main reason for X-ray use was to guide transseptal puncture.

Minimization of radiation exposure can be achieved using advanced methods such as the integration of three-dimensional electroanatomical mapping with fluoroscopy, interventional magnetic resonance imaging, and intracardiac echocardiography. However, reductions in X-ray use can also be attained via simpler practices, including reducing fluoroscopy intensity and frame rate, bringing the collimator to focus on the field of interest, keeping a distance between the operator and the X-ray tube, minimizing pedal time, and tracking operator and patient exposure levels and times.

I hope that you enjoy reading this issue of *The Journal of Innovations in Cardiac Rhythm Management*.

Sincerely,


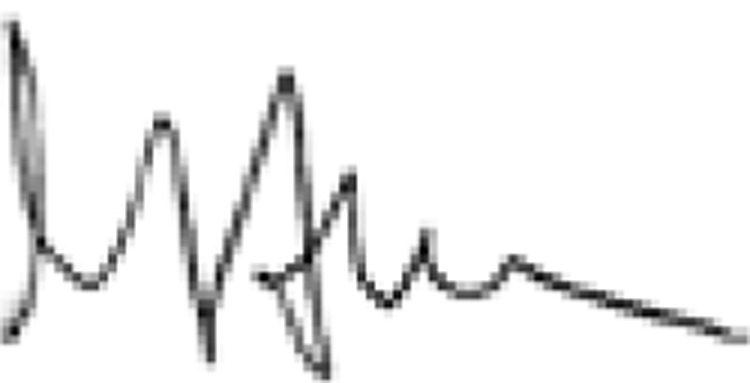


Moussa Mansour, md, fhrs, facc

Editor in Chief

The Journal of Innovations in Cardiac Rhythm Management

MMansour@InnovationsInCRM.com

Director, Atrial Fibrillation Program

Jeremy Ruskin and Dan Starks Endowed Chair in Cardiology

Massachusetts General Hospital

Boston, MA 02114
